# Intermediate addition multifocals provide safe stair ambulation with adequate ‘short‐term’ reading

**DOI:** 10.1111/opo.12236

**Published:** 2015-08-25

**Authors:** David B. Elliott, John Hotchkiss, Andrew J. Scally, Richard Foster, John G. Buckley

**Affiliations:** ^1^ Bradford School of Optometry and Vision ScienceUniversity of BradfordBradfordUK; ^2^ School of Allied Health Professions and SportFaculty of Health StudiesUniversity of BradfordBradfordUK; ^3^ Sport, Health and Performance Enhancement (SHAPE) Research GroupDepartment of Sport Science, School of Science and TechnologyNottingham Trent UniversityNottinghamUK; ^4^ Division of Medical EngineeringFaculty of EngineeringUniversity of BradfordBradfordUK

**Keywords:** multifocals, gait, falls, reading adequacy, progressive addition lenses

## Abstract

**Purpose:**

A recent randomised controlled trial indicated that providing long‐term multifocal wearers with a pair of distance single‐vision spectacles for use outside the home reduced falls risk in active older people. However, it also found that participants disliked continually switching between using two pairs of glasses and adherence to the intervention was poor. In this study we determined whether intermediate addition multifocals (which could be worn most of the time inside and outside the home and thus avoid continual switching) could provide similar gait safety on stairs to distance single vision spectacles whilst also providing adequate ‘short‐term’ reading and near vision.

**Methods:**

Fourteen healthy long‐term multifocal wearers completed stair ascent and descent trials over a 3‐step staircase wearing intermediate and full addition bifocals and progression‐addition lenses (PALs) and single‐vision distance spectacles. Gait safety/caution was assessed using foot clearance measurements (toe on ascent, heel on descent) over the step edges and ascent and descent duration. Binocular near visual acuity, critical print size and reading speed were measured using Bailey‐Lovie near charts and MNRead charts at 40 cm.

**Results:**

Gait safety/caution measures were worse with full addition bifocals and PALs compared to intermediate bifocals and PALs. The intermediate PALs provided similar gait ascent/descent measures to those with distance single‐vision spectacles. The intermediate addition PALs also provided good reading ability: Near word acuity and MNRead critical print size were better with the intermediate addition PALs than with the single‐vision lenses (*p* < 0.0001), with a mean near visual acuity of 0.24 ± 0.13 logMAR (~N5.5) which is satisfactory for most near vision tasks when performed for a short period of time.

**Conclusions:**

The better ability to ‘spot read’ with the intermediate addition PALs compared to single‐vision spectacles suggests that elderly individuals might better comply with the use of intermediate addition PALs outside the home. A lack of difference in gait parameters for the intermediate addition PALs compared to distance single‐vision spectacles suggests they could be usefully used to help prevent falls in older well‐adapted full addition PAL wearers. A randomised controlled trial to investigate the usefulness of intermediate multifocals in preventing falls seems warranted.

## Introduction

**Figure 1:**
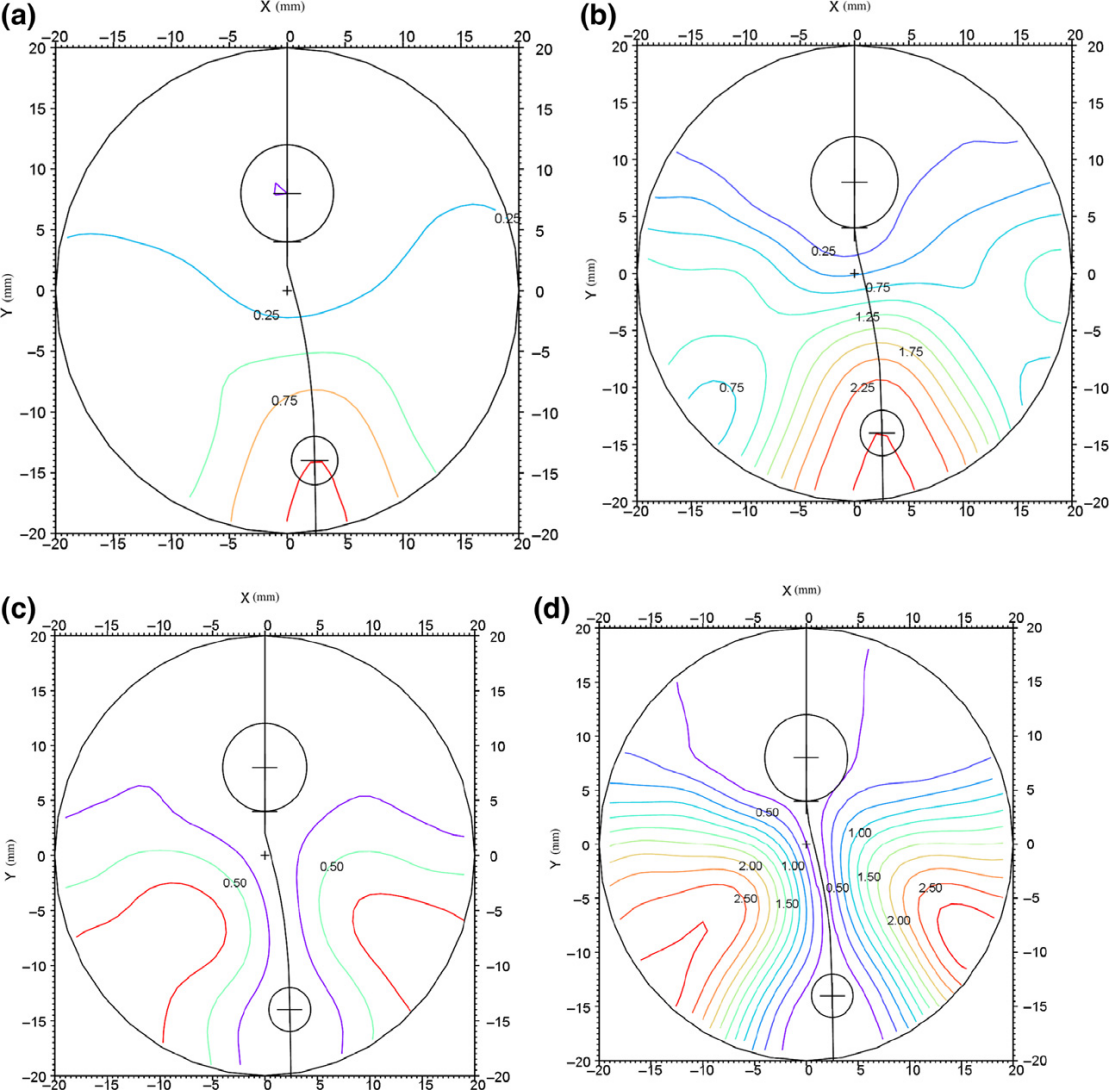
(a, b) Mean sphere (Dioptres) plots of typical progressive addition lenses with (a) a plano distance and +1.00 add, and (b) a plano distance and +2.50 add. (c, d) The PAL design also produces areas of aberrational astigmatism or distortion with (c) a plano distance and +1.00 add, and (d) a plano distance and +2.50 add. These are shown as contour plots of iso‐cylindrical lines that join points with similar amounts of surface aberrational astigmatism for the same two lenses (courtesy of Essilor International R&D).

The majority of people with presbyopia are prescribed multifocal lenses (principally Progressive Addition Lenses, PALs, or bifocals), which provide corrected distance and near vision in the same pair of glasses.^1^ However, the huge convenience of bifocals and PALs is offset to some degree by optical ‘side‐effects’: Bifocals can provide image jump as the wearer's fixation crosses the top edge of the bifocal reading section (possibly creating vertical diplopia at the dividing line^2^), and PALs provide peripheral distortion (*Figure* 1). From two walking step lengths, which is the critical distance for locating steps and obstacles at or near ground level when walking,^3^ vision is significantly worse when viewed through the near portion of high addition (~2.25–2.75 D as worn by elderly patients^1^) bifocals and PALs than through the distance portion.^4^, ^5^, ^6^ Studies suggest that long‐term multifocal wearers do not flex their heads when walking to view stairs and steps through the distance portion of their lenses,^5^, ^7^, ^8^ so that their lower visual field is typically blurred beyond about 40 cm. In a 1‐year prospective epidemiological study (*n* = 156, mean age 77 years), Lord and colleagues reported that regular bifocal/PAL wearers were more than twice as likely to fall (odds ratio 2.29, 95% confidence interval 1.06–4.92) as non‐bifocal/PAL wearers after adjusting for age and other known risk factors for falling.^4^ Bifocal/PAL wearers were also more likely to fall because of a trip, when outside their homes and on stairs. Accident data have also suggested that multifocal wear increases the risk of trips, ‘underfoot’ accidents and falls.^9^ Lab‐based studies have assessed gait safety in long‐term bifocal/PAL spectacle wearers and compared negotiating a raised surface or obstacle avoidance in bifocal/PAL vs single‐vision distance spectacles.^5^, ^6^, ^7^, ^8^ Gait adaptations included slower gait^7^, more variable toe clearance over the surface edge^5^ and ‘dropping’ on to the floor during step descent (from a raised surface/block) rather than a more controlled step down.^8^ In those studies that provide reading addition data, the average additions are 2.25 D or +2.50 D (range ~ +1.75 to +2.75 D)^5^, ^6^, ^8^ which is typical for patients of average age ~ 70–75 years^1^ as participated in those studies.^5^, ^6^, ^8^ In the other studies that have linked multifocals with falls, the average ages have been 77–80 years and patients of this age typically have reading additions of ~ +2.75 D.^1^

These studies suggested that providing long‐term multifocal wearers with a pair of distance single vision glasses for use outside the home would reduce falls risk in older frail people. This hypothesis was tested in a randomised controlled trial (RCT) of approximately 600 long‐term bifocal/PAL wearers of mean age 80 years.^10^, ^11^ Participants in the intervention group were advised to wear the distance single vision glasses when walking outside the home, other than when selecting items at the supermarket. Participants were provided with a glasses‐cord and/or a spectacle case to help with the swapping of glasses and were given verbal and written advice regarding when to wear the new glasses and why wearing the new glasses was important in terms of safety. The control group continued to use their bifocal/PAL spectacles for all tasks. Pre‐planned subgroup analysis^10^ between active and non‐active participants found a decreased falls rate for active participants in the intervention group (52%) compared to the control group (60%).^11^ In the active participants, outdoor falls and injurious falls were also less in the intervention group (42% vs 51% and 38% vs 47%).

A major problem highlighted in this RCT was trying to persuade long‐term multifocal wearers to use the distance single vision glasses when outside their home. In all, 357 people declined participation in the Haran study after initially expressing an interest in taking part and one of the reasons was that they thought that switching between two pairs of glasses required too much effort.^11^ Only 41% of participants in the study reported satisfactory adherence to wearing the additional glasses for the majority of the study (10–12 months), with 32% reporting giving up within the first 3 months.^11^ In addition, unlike other RCTs of optometric interventions,^12^ very few of the control group (2 of 301, 0.7%) were tempted to try the intervention in the follow‐up period.

In the present study we assessed whether intermediate addition multifocals could provide gait safety on stairs similar to that when using distance single vision lenses, whilst also providing adequate ‘short‐term’ reading and near vision for long‐term multifocal wearers. With an intermediate add of about 1.00–1.25 D, the floor and steps/stairs would be clear from 0.80 m to 1.0 m away and image jump or diplopia in bifocals and peripheral distortion issues in PALs would be reduced (*Figure* 1), so that gait on steps and stairs should be safer and falls risk reduced compared to when wearing full addition multifocals. We theorised that long‐term multifocal wearers would be less resistant to using an intermediate bifocal/PAL when walking outside (rather than a pair of distance single vision spectacles) as it would likely still be possible to read for short term tasks such as checking/reading the time, menus, shopping bills etc., and less switching of glasses would be required.

To assess the relative merits of using PALs/bifocals with an intermediate addition, we assessed gait when participants completed stair negotiation trials wearing these lenses compared to wearing single vision distance lenses, which we have previously found to provide safer and/or more controlled gait than multifocal lenses of the same type when ascending or descending steps of various heights.^5^, ^6^, ^8^ In order to gauge the usefulness of an intermediate addition for everyday tasks, we also tested their near visual acuity and reading speed whilst wearing the PALs with the intermediate addition.

## Methods

### Participants

Clinic records from the University of Bradford Eye Clinic were searched and letters of invitation sent to people who potentially conformed to inclusion and exclusion criteria. In addition, participants were recruited via advertisements to University staff. We originally intended to recruit both long‐term PAL and bifocal wearers, similar to previous studies,^5^, ^6^, ^8^ but only received responses from three long‐term bifocal wearers and only one of these satisfied the inclusion and exclusion criteria (data not included here). Fourteen healthy elderly long‐term PAL wearers, mean ± 1 SD age 65.2 ± 4.0 years (height 1.63 ± 0.08 m, weight 68.0 ± 11.8 kg) formed the study group. Inclusion criteria included being older than 60 years, a full‐time and current wearer of PALs (or bifocals) for at least 12 months with a near addition in both eyes of 2.00 D or more, no change in the refractive correction in the last 6 months (to ensure they were fully adapted to the lenses), a binocular visual acuity of 0.20 logMAR (Snellen 6/9 or 20/30) or better, being independently mobile and able to undertake all aspects of the experiment, anisometropia 1.50 D or less, astigmatism 1.25 DC or less and spherical refractive error between −0.75 and −4.00 DS and +0.75 and +4.00 DS. Participants with a medical history of peripheral neuropathy, rheumatoid arthritis, knee or hip replacement surgery, poor balance, or gait problems were excluded from the study as were any that had undergone cataract or any other ocular surgery in the previous 6 months, had binocular vision problems (strabismus, diplopia, amblyopia), current ocular pathology or were taking any medications that could affect vision and balance. The tenets of the Declaration of Helsinki were observed and the experiment gained ethical approval from the University of Bradford's ethics committee, with written informed consent being obtained from all participants. At the end of the study, the intermediate addition PALs were provided to each participant as compensation for participation in the study.

### Spectacle conditions

Foot clearance when negotiating a flight of stairs was assessed under six separate visual conditions which included the participant's own PAL spectacles and the following five pairs of spectacles (different styles and sizes were given to individual participants to ensure they were well fitted, but each participant had the same style and size of frame for all five conditions so that differences between conditions were due to the lens type only) and same distance refractive correction as the habitual correction:

1. PALs with the participants' full reading addition.
2. PALs where the addition was reduced by 1.50 D.
3. Bifocals with the participants full addition.
4. Bifocals where the participants' addition was reduced by 1.50 D.
5. Single vision distance lenses.

All lenses, apart from the participants' own spectacles, were Ormix 1.60 and anti‐reflection coated and included a Varilux Comfort^**®**^ New Edition Ormix^**™**^ PAL, a 28 Curved Top bifocal and distance single vision SV 360° lenses and were provided by Essilor International. All PALs were fitted with the fitting cross alignment at the centre of the pupil in primary gaze and the top of the bifocal segments were aligned with the lower lid of the participant. For each individual participant, the spectacles were fitted by an experienced optometrist to give a similar back vertex distance and pantoscopic angle as their own spectacles. All spectacles were checked by focimeter to ensure they were within international standards.

### Vision assessment

Binocular distance visual acuity was measured with the participant's habitual spectacles using an ETDRS chart at 4 m with a chart luminance of 160 cd m^−2^, using a by‐letter scoring rule and a termination rule of 4 out of 5 letters incorrect.^13^ Binocular near vision measurements were made with the participant's own PAL spectacles, the PAL with an intermediate addition, and the distance single vision lenses, in a random order. To avoid overburdening participants, we did not replicate near vision measurements with the full addition PALs or the full and intermediate bifocals as there seemed no reason why they would provide additional information. Binocular near visual acuity was measured using Bailey‐Lovie near charts at 40 cm with a chart illuminance of 400 lux, using a by‐word scoring rule. Binocular critical print size and reading speed was measured at 40 cm with MNRead charts at a chart illuminance of 400 lux using the measurement procedure indicated by the manufacturers. Three Bailey‐Lovie and three MNRead charts with different words were used in random order to avoid any memorisation effects.

### Gait assessment

**Figure 2:**
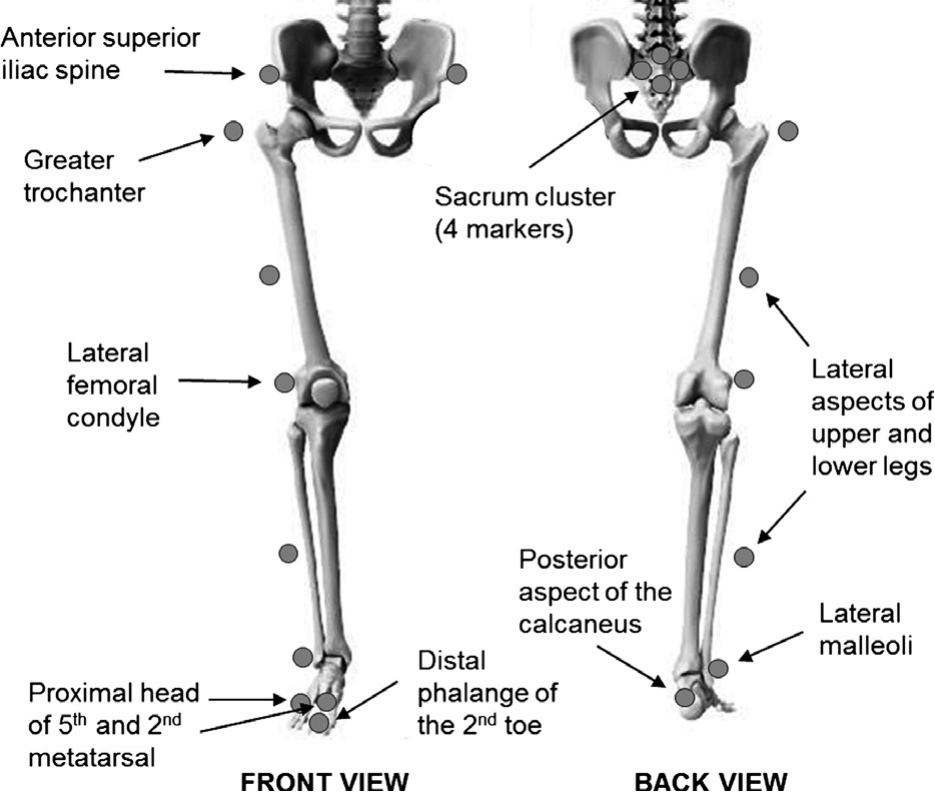
Marker positions used to determine gait kinematics during stair ascent and descent.

Vision is particularly critical for stair negotiation in older adults,^14^, ^15^ so that assessment of gait on stairs seems an appropriate assessment of vision interventions to prevent falls. Data were collected for each participant over a single 2‐h testing session. Participants were instructed prior to the session to bring a pair of shorts, t‐shirt and low‐heeled shoes and these were worn throughout the study. A full body marker set (excluding upper limbs) was used to capture segmental kinematics during stair ascent and descent using a ten camera video motion capture system (www.vicon.com) at 100 Hz. Markers were placed, in accordance with Vicon's Plug‐in Gait guidelines on the left and right anterio‐ and posterio‐ lateral aspects of the head, trunk (sternum), lateral aspect of upper and lower legs, lateral femoral condyles and lateral malleoli, and feet (posterior aspect of the calcaneus, proximal head of fifth and second metatarsals, and distal phalange of the second toe). Additional markers were placed on the lateral part of each thigh at the height of the hip centre (i.e. on the greater trochanters), and a cluster of four markers were placed on the sacrum (*Figure* 2). A digitizing wand (www.c-motion.com) was used to determine virtual landmarks that represented the stair edge locations, and to determine the anterio‐ and posterio‐ inferior points of each shoe (toe‐ and heel‐tips, respectively).

**Figure 3:**
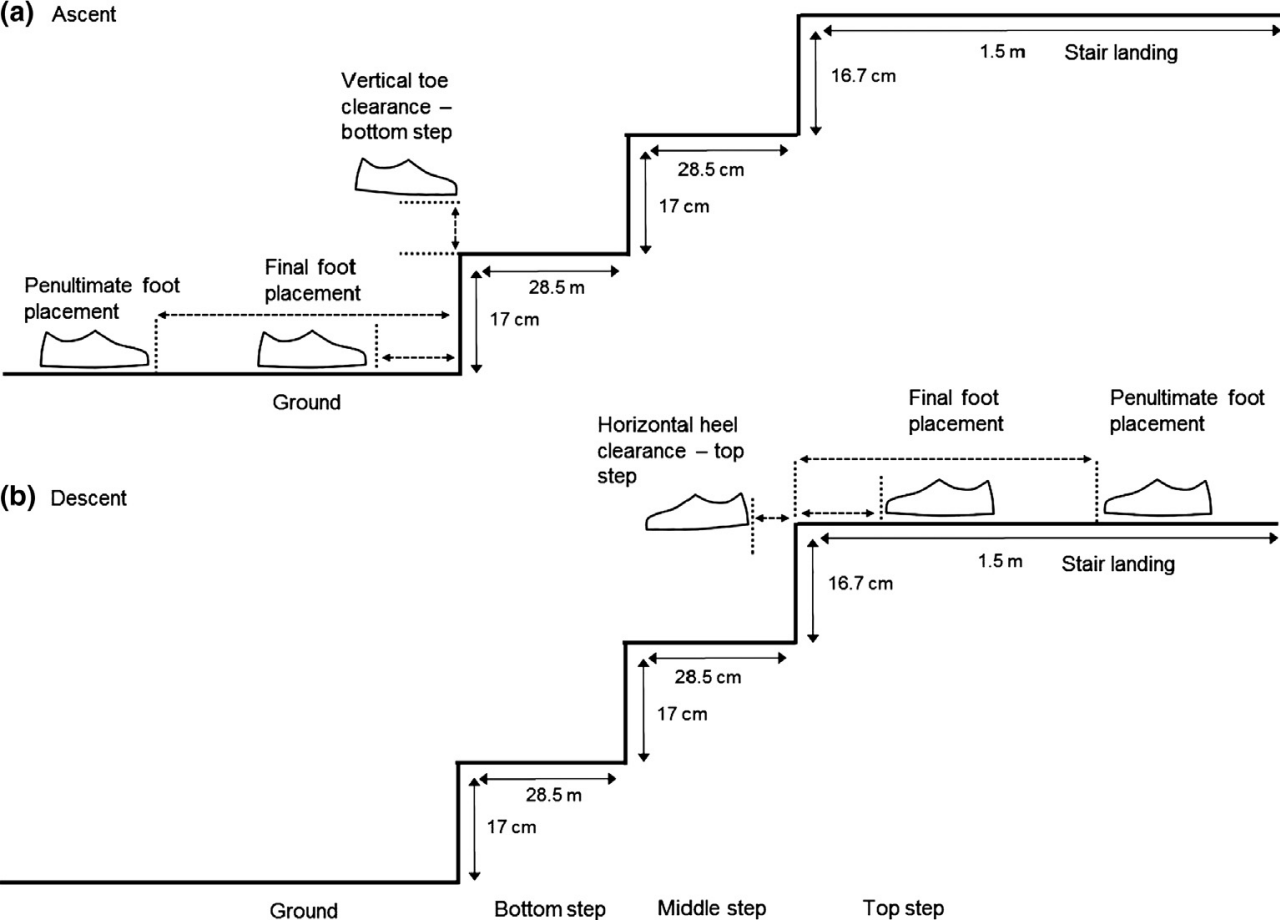
Stair dimensions and the kinematic gait variables assessed for (a) stair ascent and (b) stair descent.

The stair negotiation trials involved both stair ascent and descent (performed consecutively). The staircase consisted of three 100 cm wide steps, with a top platform area of length 150 cm (*Figure* 3)^16^. The treads of the two steps were 28.5 cm in length. The stair risers were approximately 17 cm, resulting in a stair angle of about 31°. A handrail was positioned on the right side of the staircase for safety during stair descent and crash mats were positioned to the front and left side of the staircase. The laboratory was well lit with an ambient illuminance of 400 lux.^16^

From a stationary starting position on the ground in front of the staircase, participants were provided with the first randomly allocated spectacle condition and then took two walking steps before ascending the staircase, leading with the same foot on the first step for every trial. Participants were instructed to come to a halt at the top of the stairs. After a short pause participants then walked to the end of the stair platform. The spectacles were changed and participants then turned around in preparation for descending the stairs. Stair descent was completed in a similar way to ascent: starting from two walking steps away and coming to a halt/pause after stepping on to the ground at the bottom of the staircase. An experimenter was nearby during data collection and participants were allowed to use the handrail if they felt unstable at any time during the trial.

Once participants completed the descent, they walked up and down a series of wooden stepping blocks via a range of different routes (random) in an attempt to prevent them from becoming too familiar with the riser heights used on the stairway. Five blocks of varying widths and heights were used and were arranged in a line in a separate part of the lab.

Participants were advised that the height and appearance of the stair risers would change between some trials and every fourth trial (dummy trial) the height of one or two steps were changed by adding a board (5 mm thick) to the tread. Data from these trials were not collected. This, along with the varying height block walking (following each stair negotiation trial), was undertaken to minimize learning effects whereby participants became more reliant on using proprioception during the testing session rather than using vision.^17^

Participants completed the stair negotiation trials wearing each of the six different spectacles. Trials were repeated three times for each spectacle condition in (trial‐by‐trial) random order, giving a total of 23 ascent‐descent trials including 5 dummy trials.

### Data analysis

Marker trajectories were labelled in Vicon Nexus (http://www.vicon.com/software/nexus) and the resultant C3D files uploaded to Visual 3D (http://www.c-motion.com/products/visual3d/) for further analysis. Existing stair ascent and descent marker‐based event detection algorithms were used to determine instants of foot‐contact and foot‐off in each trial.^18^ Trials were completed using a ‘step‐over‐step gait’, thus the ‘leading limb’ on each staircase step alternated between left and right limbs. As transitions from stair to floor have been shown to involve slightly different limb kinematics,^16^ heel clearance for the bottom step in descent was not assessed.

In an attempt to determine the effects of spectacle type on stair ambulation the following key variables were determined.

#### Vertical toe clearance (TC)

the mean (across repetitions) vertical distance between the leading‐limb toe‐tip (on ascent) and tread of each step at the instant the leading‐limb toe‐tip was directly above the front edge of the step. Toe clearances that are very small increase the risk of tripping^5^, ^6^, ^15^ and toe clearances that are overly large increase the time in single support (linked with a slowing of the ascent) and potentially decrease dynamic postural stability.^19^

#### Horizontal heel clearance (HC)

The mean (across repetitions) horizontal distance between the leading‐limb heel‐tip (on descent) and riser of each step as the leading‐limb heel‐tip was horizontally in line with the top edge of the step.^8^ Decreases in heel clearance lead to a greater possibility of heel scuffs/contacts and potential falls.^14^ and increases in heel clearance may lead to poor foot placement on the step below whereby the toe region of the foot overhangs the step edge and the base of support (contact area of the foot) is reduced^16^

#### Toe and heel clearance variability

The inter‐trial (across the three repetitions) standard deviation in vertical toe clearance during ascent and horizontal heel clearance during descent. Increases in variability in foot clearance indicates poor control and can increase the likelihood of trips particularly when mean clearances are small.^5^, ^6^

#### Ascent/Descent duration

Mean duration (across repetitions) from the instant of leading‐limb foot‐off prior to stepping onto the first step to the instant of leading‐limb foot‐contact on the stair landing (in ascent) or ground (in descent).^19^ An increased duration is a useful global indication of a cautionary approach as trips are less likely to lead to falls if forward movement is slower.^20^ However this may not be necessarily safer as an increase in single support time can lead to decreased dynamic postural stability.^19^

### Statistical analysis

Vision data were compared using paired 2‐tailed t‐tests in Microsoft Excel for Mac 2011 (version 14.4.1). Gait data were normally distributed and were assessed using a random effects repeated measures regression model with Maximum Likelihood estimator, using Stata Release 13.0 (www.stata.com). Differences between spectacle types were assessed using Wald χ^2^ analyses having considered the effects of stair number, where relevant (bottom, middle and top) and repetition (trials one, two and three) and any interactions. Level of significance was set at *p* < 0.05.

## Results

Participants consisted of seven hyperopes and seven myopes (median +0.88, range +2.87 to −4.12 DS) with median full and intermediate additions of +2.50 and +1.00 D (ranges +2.25 to +2.75 D and +0.75 to +1.25 D). Mean distance binocular visual acuity was ‐0.07 ± 0.07 logMAR (Snellen 6/5 or 20/17). Binocular near word acuity was significantly better with the participants' habitual PALs (mean 0.05 ± 0.09 logMAR, ~N3.5) than with the intermediate addition PALs (mean 0.24 ± 0.13 logMAR, ~N5.5; paired two‐tailed *t*‐test, *p* < 0.0001), which was better than with the distance single vision lenses (mean 0.43 ± 0.11 logMAR, N8.5; paired two‐tailed *t*‐test, *p* < 0.0001). MNRead critical print size was significantly better with the participants' habitual PALs (mean 0.13 ± 0.07 logMAR, ~N4.5) than with the intermediate addition PALs (mean 0.36 ± 0.07 logMAR, ~N7; paired two‐tailed *t*‐test, *p* < 0.0001), although reading speed above this print size was the same with both spectacles (155 ± 11 wpm vs 163 ± 18 wpm respectively, paired two‐tailed *t*‐test, *p* = 0.16).

Means and SDs for the important gait safety parameters are shown in *Table* 1 for full and intermediate addition bifocals, full and intermediate addition PALs, and distance single vision lenses. The habitual correction results (i.e. with participants' own spectacles) were similar to the results using the single vision lenses (and intermediate PALs) for all parameters (*p* > 0.10) and are not shown. It was evident during data collection that the participants were immediately aware when they were fitted with their own spectacles and they may have subconsciously acted differently (more confident and/or more relaxed?) in their own spectacles, so that comparisons with the gait data from the habitual PALs may be somewhat limited.

**Table 1:** Group mean (±1 SD) gait parameters for stair ascent (bottom stair only) and stair descent (top stair only): effects of manipulating spectacle condition

	**Bifocal**		**PAL**		**Comparison**
	**Full**	**Intermediate**	**Full**	**Intermediate**	**Single Vision**
Ascent					
Vertical toe clearance (cm)	6.9 ± 1.5	6.0 ± 1.8	6.9 ± 1.6	6.1 ± 1.6	6.2 ± 1.9
Vertical toe clearance variability (cm)	1.2 ± 0.7	0.7 ± 0.7	1.1 ± 0.6	0.8 ± 0.6	0.8 ± 0.4
Duration (s)	2.11 ± 0.38	1.98 ± 0.26	1.96 ± 0.23	1.92 ± 0.26	1.91 ± 0.27
Descent					
Horizontal heel clearance (cm)	5.4 ± 1.8	6.1 ± 1.4	7.1 ± 1.2	7.4 ± 1.7	7.2 ± 1.9
Horizontal heel clearance variability (cm)	1.5 ± 1.1	1.3 ± 1.0	1.3 ± 0.9	1.3 ± 0.6	1.2 ± 0.6
Duration (s)	2.70 ± 0.53	2.57 ± 0.49	2.49 ± 0.55	2.32 ± 0.50	2.49 ± 0.55

There were significant stair number effects for vertical toe clearance during ascent, which was reduced from the bottom to middle stair by 1.29 cm (Wald χ^2^ = 84.0, *p* < 0.0001) and from middle to the top stair by 0.36 cm (Wald χ^2^ = 6.4, *p* = 0.011). Vertical toe clearance variability was also reduced from the bottom to middle stair (Wald χ^2^ = 8.8, *p* = 0.003), but not from middle to the top stair (Wald χ^2^ = 1.0, *p* = 0.32). Horizontal heel clearance during descent was also reduced from the top to the middle stair by 5.7 cm (Wald χ^2^ = 7.8, *p* = 0.005), although its variability did not change (Wald χ^2^ = 0.2, *p* = 0.65). There were no significant spectacle by stair number interaction effects for any of the parameters analysed. Due to the lack of any spectacle by stair number interaction effects, and to simplify the results presentation, the heel and toe clearances values presented in *Table* 1 are those just for the first stair encountered: the top (for descent) or the bottom (for ascent) stair.

### Full vs intermediate multifocals

There were significant differences in the following gait safety parameters between full and intermediate bifocals (see *Table* 1): vertical toe clearance on ascent (Wald χ^2^ = 6.9, *p* = 0.009) and its variability (Wald χ^2^ = 12.0, *p* = 0.0005), ascent duration (Wald χ^2^ = 12.7, *p* < 0.0001) and heel clearance on descent (Wald χ^2^ = 8.7, *p* = 0.003). There was a significant difference between full and intermediate PALs for vertical toe clearance on ascent (Wald χ^2^ = 8.7, *p* = 0.003) only.

### Intermediate PALs vs distance single vision lenses

Gait safety parameters were similar between intermediate PALs and distance single vision lenses for all gait safety parameters measured: vertical toe clearance on ascent (Wald χ^2^ = 0.4, *p* = 0.54) and its variability (Wald χ^2^ = 0.1, *p* = 0.93), ascent duration (Wald χ^2^ = 1.4, *p* = 0.25), heel clearance on descent (Wald χ^2^ = 0.7, *p* = 0.42) and its variability (Wald χ^2^ = 2.4, *p* = 0.12) and descent duration (Wald χ^2^ = 1.6, *p* = 0.20). Gait safety parameters with the intermediate addition PALs were similar to those observed with the patient's own spectacles. As the participants were all well adapted PAL wearers, the intermediate bifocals not surprisingly provided gait safety parameters that were poorer than the intermediate PALs and/or distance single vision lenses (*Table* 1).

## Discussion

The intermediate addition PAL provided very good reading ability with mean binocular word acuity of 0.24 ± 0.13 logMAR (N5.5), with a mean reading speed of 163 wpm above a MNRead critical print size of 0.36 ± 0.07 logMAR (~N7). Given that most newspapers, books and other reading materials typically have a range of text from 0.2° to 2° (a lower limit of 1.4 mm at 40 cm, 0.38 logMAR, ~N8 or 1.0M; to make sure they match the range of text that can be read at maximum speed^21^) the present study's findings suggests that intermediate PALs would provide sufficient reading ability for the majority of everyday visual tasks undertaken by elderly individuals when outside (or indeed inside) the home environment.

The full addition multifocals caused potentially unsafe changes in stair negotiation compared to the intermediate multifocals as foot clearances were both reduced (descent) and increased (ascent), with increased variability on ascent showing poorer control and greater durations (ascent and descent) indicating increased caution. The intermediate PALs provided very similar gait characteristics to those with the single vision lenses. As might be expected given that the participants were well adapted PAL wearers, their gait with the intermediate addition bifocal was not as safe as with intermediate addition PALs or distance single vision lenses. However, their gait was substantially safer than with the full addition bifocals. This simple lab‐based study suggests that adaptive gait is safer and less cautious with intermediate addition PALs compared to full addition PALs for well adapted PAL wearers and that intermediate addition PALs provide sufficient vision for short‐term reading and other near tasks. They may also provide sufficient near vision to avoid non‐fall related injuries, the incidence of which were found to increase with the distance single vision intervention in the Haran and colleagues' RCT.^10^, ^11^ It is also important to note that in the Haran RCT, sedentary participants fell *more* outdoors (51% vs 36%), suggesting that the intervention generated false confidence and risk taking^11^ and/or that for those more likely to fall, the near focused region of multifocals may assist in response times and precision for grabbing a support/hand rail and avoiding a trip becoming a fall.^22^ and/or that the relatively few times that the sedentary participants ventured outdoors meant they had little chance to adapt to wearing the new spectacles. Note that swapping from a full addition PAL to distance single vision lenses when venturing outdoors and then swapping back again when returning home requires two adaptations (including magnification^23^ and vestibulo‐ocular reflex^24^ adaptations) from the wearer. An intermediate addition multifocal, which could be worn most of the time inside and outside the home and thus avoid continual switching, with a pair of full addition multifocals or single vision reading spectacles for long‐duration or concentrated near tasks (while stationary) may be preferred.

Limitations of this study include the lack of participants habitually wearing bifocals for comparison. It is likely that the percentage of bifocal wearers in the wider population is getting smaller while PAL provision is increasing. For example, in the 2002 Australian epidemiological study by Lord, 87% of the multifocal wearing participants wore bifocals,^4^ while in their RCT with recruitment between 2005 and 2007, 60% wore bifocals.^10^, ^11^ Similarly, in our 2007 UK lab‐based study, 12 of 19 participants (63%) were bifocal wearers,^5^ yet we had great difficulty recruiting bifocal wearers for this study. Another limitation was the inclusion of the relatively high number of spectacle conditions which may have led to a repeated gait pattern being used across trials, which may have reduced the sensitivity of the gait assessments to detect differences between single vision and intermediate add PAL lenses. We attempted to avoid participants adopting a repeated (automated) gait pattern by using different starting points and using dummy trials with different heights of stair. The fact that differences in gait pattern for different spectacle types were found suggests that we were at least partly successful in this, but relatively subtle differences in gait between single vision spectacles and intermediate PALs may have been missed.

### Summary/Conclusions

A recent randomised controlled trial indicated that falls rate can be reduced in active, long‐term multifocal wearers if they wear distance single vision lenses outdoors.^10^, ^11^ However, compliance with this intervention was poor.^11^ In the present study, the safer gait parameters for the intermediate addition compared to the full addition multifocals plus the similarity in gait with the single‐vision spectacles (for the intermediate addition PALs) suggests that their use outdoors should provide similar gait safety to distance single vision spectacles for active long‐term PAL wearers. In addition the good short term reading ability with the intermediate addition PALs compared to single‐vision spectacles suggests that elderly individuals would be more likely to comply with the use of intermediate addition PALs outside the home. The cost of an intermediate add pair of PALs plus a pair of single vision reading spectacles would be the same as a full addition pair of PALs plus a pair of single vision distance spectacles as recommended by Haran and colleagues^11^; it would represent a greater cost if an intermediate pair of PALs was coupled with a full addition PAL for reading tasks and both the recommendations here and from Haran *et al*.^11^ represent a greater cost than one pair of PALs. However, this should be considered in comparison to the cost of falls to both the Health Service^25^ and the morbidity of the patient.^26^ We suggest that a randomised control trial is required to determine whether trips and falls incidences are reduced as a result of using such spectacles when walking outside and whether compliance is better than switching to distance single vision spectacles.

## Disclosure

Essilor International R&D were involved in determining the inclusion and exclusion criteria for the study and required that a detailed head position system was used to provide details regarding the position of centre of rotation of the eyes relative to the freeform lenses (this was of no significance for this particular study and so these data were not reported).
